# Eye care where there are no ophthalmologists: the Uganda experience

**Published:** 2020-12-31

**Authors:** Joseph Magyezi, Simon Arunga

**Affiliations:** 1 Ophthalmic Clinical Officer: Ruharo Eye Centre, Ruharo Mission Hospital, Mbarara, Uganda.; 2 Consultant Ophthalmologist: Mbarara University of Science and Technology, Mbarara, Uganda.

**Ophthalmic clinical officers (OCOs) in Uganda undergo a year of intensive training; this qualifies them to provide high quality eye care in a country affected by a shortage of ophthalmologists.**

**Figure:**
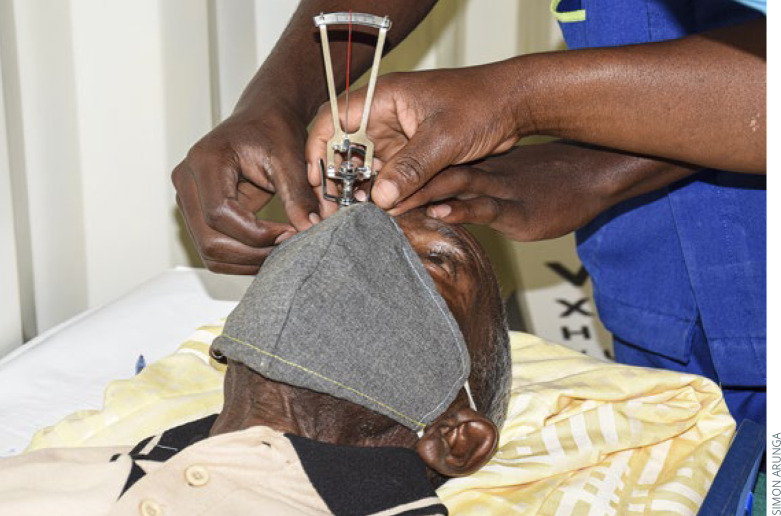
An ophthalmic clinical officer, supported by an ophthalmic assistant, measures intraocular pressure using a Schiotz tonometer. Both patient and eye health workers are wearing masks to reduce the risk of COVID-19 transmission. **UGANDA**

A shortage in human resources for eye health is a huge problem in many parts of sub-Saharan Africa, with most countries failing to achieve the ratio of 4 ophthalmologists per million population recommended by the World Health Organization in 2006.^1^,^2^ Uganda's population of 40 million is served by just 40 ophthalmologists (1 per million population), and half of them work in the capital city, Kampala. This means that the majority of eye care services in the country are provided by non-ophthalmologists.

In Uganda, we have allied eye health personnel known as ophthalmic clinical officers (OCOs). To become an OCO, a nurse (with a diploma in nursing from a registered institution) or medical clinical officer (with a diploma in clinical medicine) must complete a diploma in clinical ophthalmology, a one-year full-time course offered by the Ophthalmic Clinical Officers Training School Uganda. The training school was established in 1989 and is based in Jinja.

**“The majority of eye care services in Uganda are provided by non-ophthalmologists.”**

Upon completion of the training, OCOs work at the existing primary health centres in each catchment area. Here, they are responsible for establishing and planning eye care services for the local population. OCOs manage common eye diseases and conditions, organise eye camps to visit rural areas, refer patients who need surgery, and manage eye care equipment and consumables. OCOs also run the eye health management information system (HMIS). They make daily entries in their eye clinic registers and, once a month, enter a summary of this information into the HMIS system. These data are then aggregated with other health unit data, submitted to the district information office, and reported to the Uganda Ministry of Health.

**From the editor**

Read more about OCOs in Uganda in a previous issue of this journal: **bit.ly/ugandaOCO**

In this article, we draw from our Ugandan experience to provide guidance on how OCOs, or similarly qualified allied eye health personnel, can effectively provide eye care services at primary health centres in communities where there are no ophthalmologists.

## Setting up an eye clinic: practical considerations

OCOs must be able to test visual acuity, carry out basic evaluations, and perform simple procedures.

### The space

The clinic should have a well-lit room suitable for testing visual acuity (VA). Ideally, the room would be 6 metres in length. However, most primary health centres do not have a designated eye unit and may not have such a room. A 3-metre room can be adapted by placing the VA chart above the patient and a mirror on the opposite side of the room. The patient is then asked to read the letters through the mirror, thereby doubling the visual distance to 6 metres. Whilst not ideal, as there is no control of luminance, a VA chart can be placed on a wall outside the clinic room, and patients can stand 6 metres away in the compound to read. **Note:** Where possible, ensure that patients are standing in the shade when looking at the chart; bright sunlight can cause glare and may affect the test results.

### Equipment

- **Visual acuity testing charts.** The Snellen E chart or the tumbling E chart is used most often. These are very useful, especially in a rural population where the majority of people are unable to read.
- **Source of light (torch).** A torch is a useful tool for basic eye examination, pupil assessment, and identification of many ocular pathologies. We have recently advocated for torches which have a blue light option to aid in the diagnosis of corneal abrasions and microbial keratitis.^3^
- **Magnifying loupes.** These aid in visualising finer details on the eye; for example, when identifying or removing foreign bodies. If you do not have loupes, you can use simple reading spectacles to magnify the ocular structures.
- **Direct ophthalmoscope.** This is a useful tool for examining the deeper structures of the eye, such as the retina, optic nerve, and macula. It can also be used to identify cataract, especially a posterior subcapsular cataract which is not easily identifiable on torch examination. There are now cheaper options like the Arclight (see **bit.ly/CEHJarc**), which has many additional benefits such as magnification, blue light and solar charging.
- **Tonometer.** This is useful for measuring intraocular pressure (IOP). Although the Goldman tonometer, mounted on a slit lamp, is the ‘gold standard’ for IOP testing, and newer technology such as the icare tonometer is well tolerated across many age groups, they are both expensive and not readily available in many parts of Uganda. Cheaper tonometers, such as the Shiotz tonometer, are a useful and reliable alternative.
- **Pharmaceuticals.** These are important for diagnosis and simple treatment procedures. They include local anesthetic, mydriatics, and flourescein strips. Fluorescein strips can be made using locally sourced ingredients. Further information on how to prepare fluorescein strips has recently been published in this journal (**bit.ly/CEHJfluor**).^4^
- **A trial lens set and frame.** This enables simple subjective refraction. A modest number of OCOs in Uganda (30%) have received additional training in refraction and can perform refraction assessment for simple uncorrected refractive errors and presbyopia.

**COVID-19 precautions**

In light of the COVID-19 pandemic, it is now recommended to take precautions when carrying out procedures that may bring you in close contact with patients. We recommend making and using simple face shields or visors (see **bit.ly/CEHJvisor**) and wearing surgical masks, if available, to avoid droplet transmission. Keep windows open to improve ventilation. The eye care practitioner and patient should avoid speaking while the eye examination is carried out.

### How to manage the day-to-day running of the clinic

It is important to think about efficiency, good use of resources, space, and the quality of patient care.

#### Clinic days

In most primary health centres, the eye unit space is shared with other disciplines such as ear, nose and throat (ENT) clinics, orthopaedic clinics, and others. It is important to ensure consistency of service, e.g., having the clinic on the same day and at the same time every week (or fortnight) and never missing one, as this helps to drive demand and uptake. Depending on the volume of patients, some OCOs run weekly, bi-weekly or daily clinics.

**Figure:**
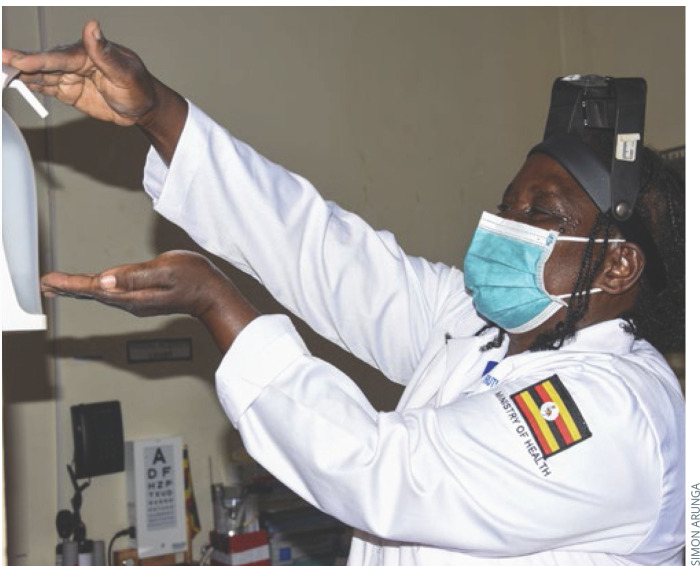
The COVID-19 pandemic has highlighted the importance of regular hand washing and hand sanitising between patients. **UGANDA**

#### Task shifting or task sharing

Ideally, the eye unit in a primary health centre will be run by one OCO supported by a nurse working as an ophthalmic assistant (see panel below); however, our recent evaluation of the health system in Uganda noted general staffing levels of about 50% in most primary health centres.^3^ Because of this, many eye units are often run by an OCO on her or his own.

**“It is important to plan task allocation carefully to maximise productivity and efficiency.”**

It is therefore important to plan task allocation carefully to maximise productivity and efficiency. This enables the OCO to focus on key eye examination tasks that cannot be shifted to, or shared with, other staff members.

Many OCOs rely on staff members from other units to provide additional support. For example, registration of patients can be done centrally by the clinic's records assistants, visual acuity testing can be done by volunteer nurses (we have trained our driver to test visual acuity during outreach activities), and pharmacy assistants can dispense eye medication. If a primary health centre has weekly eye clinics, rather than daily eye clinics, the OCO may spend the other days helping out in other clinics, such as HIV.

**Allied ophthalmic personnel**

Allied ophthalmic personnel known as ophthalmic assistants (OAs) help OCOs to run eye clinics, and ophthalmic operating theatre nurses (OTNs) provide support in the operating theatre. OAs and OTNs are usually nurses with a certificate from a registered institution who undergo full-time ‘on-the-job’ training in an accredited tertiary eye hospital setting over a period of three months. These staff members are not officially recognised in the general health system structure of Uganda and are not permitted to work independently.

#### Comprehensive assessment

It is important to realise that the primary health centre is usually the first point of contact with the health system. Assessment should be tailored to be as comprehensive as possible in order to identify conditions without obvious symptoms - such as glaucoma, diabetic retinopathy and uncorrected refractive errors - in line with local needs. In our area, for example, we routinely assess people over 35 years of age for glaucoma. In a recent meeting with OCOs in southwestern Uganda, however, only 10% reported that they were routinely assessing people for glaucoma, indicating potential room for improvement.

### Staying in touch

Good communication with the nearest referral centre or ophthalmologist is important for several reasons:

- **It facilitates referral.** In Africa, the referral system is not straightforward, and many patients do not eventually go when referred. Reasons could include transportation costs, the distance they have to travel, the need for someone to accompany them and fear of the unknown. Generally, it is good for OCOs to be aware of where the nearest ophthalmologist is and which eye conditions they are able to manage. For example, although the secondary hospital may be in the next town, and the retinal surgeon further away, you may want to send a patient directly to the retinal surgeon if that will save time and cost. Further information on referral, with particular reference to microbial keratitis, can be found in a previous article in this journal.^5^
- **It makes teleconsultation possible.** In many parts of Uganda, there is good mobile phone coverage. Where there is a good relationship between an OCO and an ophthalmologist, the OCO can take a photograph and send it directly to the ophthalmologist if a second opinion is needed, which is a great resource. However, some of the finer details of the eye may not be easily captured with a smartphone camera. For this reason, we are piloting the use of macro lens phone adaptors to better capture corneal images for teleconsultation.
- **It allows counter-referral (from the hospital back to primary level).** One of the main challenges with eye health care in Africa is loss to follow-up. For example, only 30% of patients who receive cataract and glaucoma surgery in our hospital return at six weeks. If these patients are referred back to the primary health centre for follow-up instead (known as counter-referral), a greater proportion of patients will be able to attend as the primary health centre is closer to their homes. The referral and counter-referral mechanism used in Uganda also supports OCOs in their continuous professional development (CPD). This is because the Ministry of Health referral forms include space for the ophthalmologist to provide feedback to the referring OCO, indicating the findings, diagnosis and management plan. Many OCOs find this useful as a way of learning, and it helps communication to flow both ways.
- **It provides opportunities for offering outreach services.** Proactive OCOs can organise outreach services in their facilities. For example, a mobile team from the secondary facility can come directly to the primary health centre to provide cataract surgery. This helps to build trust in the primary health centre and creates demand for eye services in the community, all of which helps to increase the number of patients who come to the centre.

**Figure:**
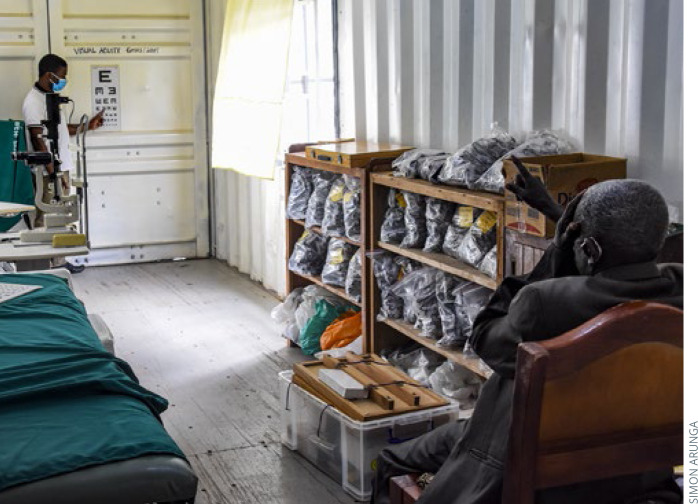
An ophthalmic clinical officer measures visual acuity. **UGANDA**

### Clinical considerations

The services provided at the primary health centre are predominantly screening, treatment and referral; most centres offer the first level of care for the majority of patients. Our recent work found that patients present quite early to these primary health centres,^6^ which provides an opportunity to provide early treatment, referral and health promotion messages.

At the primary health centre, it is important to ask two important questions to guide the decision-making process, particularly with regards to referral:

1. Is this condition urgent, or not?
2. Is this condition manageable at this level, or not?

For further information regarding the management of eye conditions, including eye emergencies, please see previous issues of this journal, such as **cehjournal.org/managing-and-preparing-for-eye-emergencies**.

### Guidance and second opinions

Many primary health centres in Uganda have a copy of the National Treatment Guidelines for eye care provided by the Uganda Ministry of Health (available from **www.health.go.ug/cause/ministry-of-health-guidelines-for-eye-care**). This document helps to inform the timing of referrals and the most appropriate treatment modalities. In addition, mobile teleconsultation with an ophthalmologist at the secondary level is widely practiced (as described above) whenever there is a query regarding diagnosis or appropriate treatment regimes.

### Keeping up to date

Many parts of Uganda have decent internet coverage and most eye care professionals can access free online resources, such as this journal. The Ophthalmology Society of Uganda also organises seminars periodically.
